# The Accuracy of Sepsis Screening Score for Mortality Prediction at Emergency Department Triage

**DOI:** 10.5811/westjem.2022.6.56754

**Published:** 2022-08-11

**Authors:** Karn Suttapanit, Sirasit Satiracharoenkul, Pitsucha Sanguanwit, Thidathit Prachanukool

**Affiliations:** Mahidol University, Faculty of Medicine Ramathibodi Hospital, Department of Emergency Medicine, Bangkok, Thailand

## Abstract

**Introduction:**

Sepsis has a mortality rate of 10–40% worldwide. Many screening tools for sepsis prediction and for emergency department (ED) triage are controversial. This study compared the accuracy of the scores for predicting 28-day mortality in adult patients with sepsis in the triage area of the ED.

**Methods:**

Adult patients who presented to the ED of a tertiary-care university hospital from January–December 2019 with an initial diagnosis of sepsis or other infection-related conditions were enrolled. We calculated predictive scores using information collected in the ED triage area. Prognostic accuracy was measured by the area under the receiver operating characteristic curve (AUROC) for predicting 28-day mortality as a primary outcome. The secondary outcomes included mechanical ventilation usage and vasopressor usage for 28 days.

**Results:**

We analyzed a total of 550 patients. The 28-day mortality rate was 12.4% (n = 68). The 28-day mortality rate was best detected by the National Early Warning Score (NEWS) (AUROC = 0.770; 95% confidence interval [CI]: 0.705–0.835), followed by the quick Sequential Organ Failure Assessment (qSOFA) score (AUROC = 0.7473; 95% CI: 0.688–0.806), Search Out Severity (SOS) score (AUROC = 0.749; 95% CI: 0.685–0.815), Emergency Severity Index (ESI) triage (AUROC = 0.599; 95% CI: 0.542–0.656, and the Systemic Inflammatory Response System (SIRS) criteria (AUROC = 0.588; 95% CI: 0.522–0.654]). The NEWS also provided a higher AUROC and outperformed for 28-day mechanical ventilator usage and 28-day vasopressor usage.

**Conclusion:**

The NEWS outperforms qSOFA, SOS, SIRS, and ESI triage in predicting 28-day mortality, mechanical ventilator, and vasopressor usage of a patient with sepsis who is seen at ED triage.

## INTRODUCTION

Sepsis is a clinical syndrome of life-threatening organ dysfunction caused by a dysregulated host response to infection.^1^ Over the past 30 years, sepsis has increasingly become an area of interest both in diagnosis and management because of its high mortality rate. Despite this increased focus, the mortality rate of sepsis is still high,^2^ averaging 39% worldwide.^3^ The Third International Consensus Definitions for Sepsis and Septic Shock (Sepsis-3) recommended the application of the Sequential Organ Failure Assessment (SOFA) to identify organ dysfunction or failure in sepsis patients.^1^ When SOFA was compared with the original Systemic Inflammatory Response Syndrome (SIRS) criteria, SOFA outperformed SIRS in predicting hospital mortality. The consensus suggested quick sequential organ failure assessment (qSOFA) as a screening tool in patients who are likely to have sepsis; qSOFA was proven to offer predictive validity similar to SOFA.^4^

In 2016 the Surviving Sepsis campaign recommended the implementation of sepsis screening, which has been shown to improve outcomes and reduce the mortality rate.^5^ Many predictive scores, such as the National Early Warning Score (NEWS), were developed and implemented to detect deterioration in sepsis patients.^4^ These scores can be used as a general screening tool as well as an early warning tool in the emergency department (ED), guiding collaboration with other areas in the hospital and the patient care system. The Emergency Severity Index (ESI) triage tool is a five-level ED triage algorithm that provides clinically relevant stratification of patients from 1 (the most emergent priority) to 5 (the least urgent priority) based on acuity and resource needs.^6^. However, the ESI triage tool was not specifically designed for severity classification in sepsis patients. The Search Out Severity (SOS) score was the early sepsis score used in Thailand. It has been shown that the implementation of a combined SOS score for screening with a checklist for sepsis bundles could decrease the mortality rate in Thailand.^2^

This study compares the accuracy of qSOFA, NEWS, SOS, SIRS, and ESI triage for predicting 28-day mortality in adult patients with sepsis, with the goal of designing an appropriate screening tool for use in the ED triage area.

## METHODS

### Study Design and Setting

This was a retrospective cross-sectional study. We collected data in the ED of a tertiary-care university hospital, between January–December 2019. The study was approved by the Ethics Committee of our institution.

### Study Population

The study included patients >18 years who presented to the ED with a diagnosis of sepsis or infection-related conditions (Appendix 1) and had been treated with the sepsis protocol in the ED. We enrolled patients by day and alternated the days of the ED visit to reach the calculated sample size. The exclusion criteria were patients who transferred from other hospitals or areas of the hospital and patients with incomplete 28-day follow-up data.

### Data Measurement and Outcomes

Data collection included patient demographics, presenting symptoms, vital signs recorded at the triage area, provisional diagnosis, hemoculture status, site of infection, 28-day intubation status, 28-day vasopressor time, and 28-day mortality. The variables of qSOFA, NEWS, SOS, SIRS, and ESI triage were recorded using the information gathered from the triage area of the ED (Appendix 2). The primary outcome was 28-day mortality. The secondary outcomes were mechanical ventilator usage within 28 days and vasopressor usage within 28 days.

Suspected sepsis was defined by physicians in the ED using the sepsis protocol, including qSOFA in Sepsis-3 criteria^1^ or physicians’ clinical judgment in the ED. Some physiologic parameters were not used because our goal was to compare predictive scores, which were used as a screening tool in the ED triage area. Thus, for example, the maximum score for SIRS was 3 because white blood cell count was disregarded, and the SOS score did not include urine output. Furthermore, a Barthel index of 20 was used to define totally dependent activities of daily living (ADL), and heart failure with reduced ejection function (HFrEF) was defined as a left ventricular ejection fraction of 40% on transthoracic echocardiography, which was documented in the medical records.

**Population Health Research Capsule**

What do we already know about this issue?

*Many screening tools are available at the triage area of the emergency department*.

What was the research question?

*Which triage screening tool is the most accurate for predicting mortality in patients with sepsis?*

What was the major finding of the study?

*The National Early Warning Score outperforms other sepsis screening tools (area under the receiver operator characteristic curve of 0.77) in predicting mortality, need for ventilator and vasopressors for patients evaluated at ED triage*.

How does this improve population health?

*Using the most accurate screening tool at ED triage could enhance the healthcare of the population, including patients with sepsis*.

### Sample Size and Data Analysis

We calculated the sample size for this study by using the equation N = Z_α/2_^2^p(1 − p)/d^2^, with the standard normal variate (Z_α/2_) at 5%, the probability of expected sensitivity (p) equals 0.9. A two-sided test concluded that the minimum sample size would be 139 samples. The mortality rate for sepsis is 39%, as reported in a previous study.^3^

### Statistical Analysis

We compared the survival and the nonsurvival groups by using the chi-square or Fisher’s exact test for categorical variables and the t-test for continuous variables. The data was presented as a percentage for categorical data and as a mean with standard deviation or median with interquartile range, as appropriate, for numerical data. The area under the receiver operating characteristic curve (AUROC), with a 95% confidence interval (CI), was depicted to evaluate the discrimination performance of each score. Sensitivity and specificity were calculated for each score as well. A *P*-value less than 0.05 was considered significant. We used STATA version 16.1 for statistical analysis (StataCorp LLC, College Station, TX).

## RESULTS

**Figure 1:**
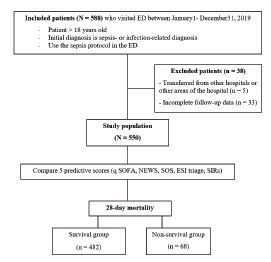
Protocol flow chart for sepsis screening study at emergency department triage. *ED*, emergency department; *qSOFA*, quick Sequential Organ Failure Assessment; *NEWS*, National Early Warning Score; *SOS*, Search Out Severity; *ESI*, Emergency Severity Index; *SIRs*, Systemic Inflammatory Response syndrome.

In total, 550 patients were included in the analysis. A protocol flow chart is shown in Figure 1. The overall 28-day mortality was 12.4%. The overall 28-day mechanical ventilator usage and 28-day vasopressor usage were 23.2% and 18.1%, respectively. The mean age was 69 years, and 46.7% of patients were male. The three most common comorbidities were diabetes mellitus (31.6%), solid-organ malignancy (25.8%), and totally dependent ADL (19.8%). The mortality was significantly higher in comorbidities such as the solid organ tumor group, the hematologic malignancy group, and HFrEF group. Vital signs such as higher heart rate (118 vs 106, *P* <0.001) and respiratory rate (27 vs 24), *P* <0.001) and lower systolic blood pressure (112 vs 106, *P* <0.001) and oxygen saturation (92 vs 96), *P* <0.014) were significant in mortality. The patient demographic data in the survival and nonsurvival groups, is summarized in Table 1.

**Figure 2:**
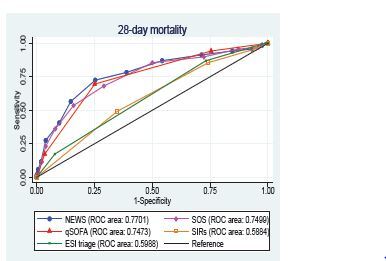
The AUROC of the predictive scores for predicting 28-day mortality. *AUROC*, Area Under the Receiver Operating Characteristic curve; *ROC*, receiver operating characteristic; *NEWS*, National early warning score; *SOS*, Search Out Severity Score; *qSOFA*, quick Sequential Organ Failure Assessment; *SIRS*, Systemic Inflammatory Response Syndrome; *ESI*, Emergency Severity Index.

The primary outcome, 28-day mortality, was best detected by NEWS (area under the receiver operating characteristic curve [AUROC] = 0.770; 95% CI: 0.705–0.835), followed by SOS (AUROC = 0.749; 95% CI: 0.685–0.815, qSOFA (AUROC = 0.7473; 95% CI: 0.688–0.806]), ESI triage (AUROC = 0.599 ;95% CI: 0.542–0.656), and SIRS (AUROC = 0.588; 95% CI: 0.522–0.654], as shown in Table 2 and Figure 2. The sensitivity and specificity for predicting the 28-day mortality rates of all predictive scores at different threshold are presented in Table 3.

**Figure 3:**
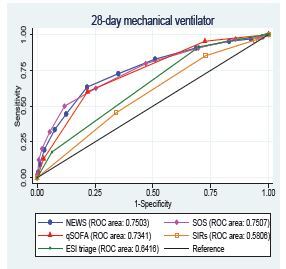
The AUROC of the predictive scores for predicting 28-day mechanical ventilator usage. *AUROC*, Area Under the Receiver Operating Characteristic curve; *ROC*, receiver operating characteristic; *NEWS*, National early warning score; *SOS*, Search Out Severity Score; *qSOFA*, quick Sequential Organ Failure Assessment; *SIRS*, Systemic Inflammatory Response Syndrome; *ESI*, Emergency Severity Index.

**Figure 4:**
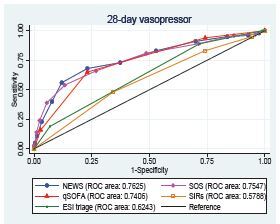
The AUROC of the predictive scores for predicting 28-day vasopressor usage. *AUROC*, area under the receiver operating characteristic curve; *ROC*, receiver operating characteristic; *NEWS*, National Early Warning Score; *SOS*, Search Out Severity Score; *qSOFA*, quick Sequential Organ Failure Assessment; *SIRS*, Systemic Inflammatory Response syndrome; *ESI*, Emergency Severity Index.

For the secondary outcomes, 28-day mechanical ventilator usage and vasopressor usage, NEWS provided a high AUROC and outperformed as shown in Table 2 and Figures 3, 4.

## DISCUSSION

Our study demonstrates that NEWS has the best predictive performance for the 28-day mortality of sepsis patients at the triage area of the ED. In the same way, Omar et al reported that NEWS outperformed both SIRS (AUROC 0.95 vs 0.89; *P* 0.001) and qSOFA (AUROC 0.95 vs 0.87; *P* 0.001) in predicting death in only the severe sepsis and septic shock groups in the ED.^7^ Anniek et al determined that NEWS performed substantially better than qSOFA and SIRS in predicting both 10-day mortality (AUROC = 0.837, 0.744, and 0.646, respectively) and 30-day mortality (AUROC = 0.779, 0.697, and 0.631, respectively).^8^ Furthermore, NEWS showed a high performance in predicting 28-day mechanical ventilator and 28-day vasopressor used. These results were in accordance with the previous study by Churpek et al,^9^ which showed that general early warning scores (EWS) are more accurate than qSOFA in predicting adverse outcomes of sepsis outside the intensive care unit setting.

In the triage area of the ED, qSOFA was easier to assess by less experienced medical professionals.^1^ However, qSOFA has a limited ability to predict poor outcomes in sepsis patients.^10^,^11^ Additionally, the metrics used in EWS are standard measures that can be readily and rapidly performed throughout the healthcare system as well as in the ED triage area. The NEWS also demonstrates a higher performance than the SOS score, which necessitates information not available in the triage area. Chompunot et al^2^ conducted a study in the hospital referral system that did not focus on the triage area. Their study found that the ESI score, which is commonly used as the general screening tool at ED triage, was inferior to NEWS, SOS, and qSOFA in predicting sepsis-related 28-day mortality, 28-day mechanical ventilator, and 28-day vasopressor used. Moreover, determining ESI at triage requires evaluator experience, as well as differing cut-off values of the parameters with other tools from other patient care systems.

Because of its strong predictive accuracy and simplicity, our findings support the use of NEWS as a screening tool in ED triage.^5^,^12^–^15^ An automatic calculation in the sepsis alert system likewise correctly uses NEWS.^16^ A NEWS cut-off prediction score of ≥ 4 (sensitivity 91.30%, specificity 28.69%) and ≥ 5 (sensitivity 86.96%, specificity 45.74%) predicted sepsis-related 28-day mortality, according to our findings. The score has the highest sensitivity (90%) and specificity (25%) for activating sepsis alarms.

## LIMITATIONS

This was a retrospective, single-center study. Second, it should be noted that substantial numbers of patients had advanced-stage malignancies, including solid organ and hematologic malignancies, which had a higher mortality rate. Additionally, patients who did not resuscitate were not excluded from our study, which could have affected the outcome.

## CONCLUSION

The National Early Warning Score outperforms qSOFA, SOS, SIRS, and ESI triage scores in predicting 28-day mortality, mechanical ventilator usage, and vasopressor usage of a patient with sepsis in the triage area of the ED.

## Supplementary Information





## Figures and Tables

**Table 1:** Baseline characteristics of patients stratified by 28-day mortality.

Characteristic	All (N = 550)	Survivor (n = 482)	Non-survivor (n = 68)	P-value
Age, year, mean (SD)	69 (16.5)	68 (16.9)	72 (12.9)	0.105
Male, n (%)	257 (46.7)	216 (44.9)	39 (57.4)	0.066
Comorbidities, n (%)				
Cirrhosis	29 (5.3)	28 (5.8)	1 (1.4)	0.692
Diabetes mellitus	174 (31.6)	155 (32.2)	18 (26.1)	0.296
Hematologic malignancy	38 (6.9)	29 (6.0)	9 (13.0)	0.033
Solid-organ malignancy	142 (25.8)	110 (22.9)	32 (46.4)	<0.001
AIDS with opportunistic infection	8 (1.5)	8 (1.7)	0 (0.0)	0.280
Transplant status	19 (3.5)	19 (4.0)	0 (0)	0.093
Immunocompromised	55 (10.0)	49 (10.2)	6 (8.7)	0.692
ESRD on RRT	51 (9.3)	47 (9.8)	4 (5.8)	0.284
COPD group D	26 (4.7)	22 (4.6)	4 (5.8)	0.661
Heart failure	23 (4.2)	13 (2.7)	10 (14.5)	<0.001
Neuromuscular disease	2 (0.4)	2 (0.4)	0 (0)	0.592
Totally dependent ADL	109 (19.8)	93 (19.3)	16 (23.2)	0.464
Chief complaint, n (%)				
Fever	294 (53.5)	267 (55.7)	27 (39.1)	0.010
Alteration of consciousness	61 (11.1)	47 (9.8)	14 (20.3)	0.010
Dyspnea	98 (17.8)	74 (15.4)	22 (31.9)	0.001
Cough	5 (0.9)	5 (1.0)	0 (0)	0.395
Malaise	7 (1.3)	7 (1.5)	0 (0)	0.313
Nausea/Vomiting	10 (1.8)	9 (1.9)	1 (1.4)	0.804
Abdominal pain	25 (4.5)	23 (4.8)	2 (2.9)	0.480
Other	50 (9.1)	47 (9.8)	3 (4.3)	0.141
Vital signs, mean (SD)				
Heart rate, per minute	107 (23.9)	106 (23.2)	118 (26.4)	<0.001
Temperature, Celsius	38.0 (3.2)	38.1 (3.0)	37.5 (4.2)	0.089
Respiratory rate, per minute	24 (4.8)	24 (4.5)	27 (5.9)	<0.001
Systolic blood pressure, mmHg	130 (33.2)	133 (31.8)	112 (37.4)	<0.001
Diastolic blood pressure, mmHg	71 (16.2)	72 (15.4)	65 (19.9)	0.001
Oxygen saturation, %	96 (7.8)	96 (7.7)	92 (8.0)	0.014
Mental status, n (%)				
Alert	404 (73.5)	367 (76.6)	35 (50.7)	<0.001
Response to verbal	88 (16.0)	71 (14.8)	17 (24.6)	0.045
Response to pain	35 (6.4)	28 (5.8)	7 (10.1)	0.135
Unconsciousness	23 (4.2)	13 (2.7)	10 (14.5)	<0.001
Venous lactate, mmol/dL, mean (SD)	2.5 (1.8)	2.3 (1.8)	3.8 (2.9)	<0.001
Disposition, n (%)				
Discharge	290 (53.1)	283 (59.5)	7 (10.1)	<0.001
General ward	144 (26.4)	132 (27.7)	12 (17.4)	0.073
Intensive care ward	78 (14.3)	56 (11.8)	21 (30.4)	<0.001
Dead at emergency department	6 (1.1)	0 (0)	6 (8.7)	<0.001
Palliative care ward	28 (5.1)	5 (1.1)	23 (33.3)	<0.001
Length of hospital stay in hours, median (IQR)	68 (11,233)	56 (11,199)	142 (63,342)	0.045
Predictive score, median (IQR)				
NEWS	5 (3,7)	5 (3,7)	8 (6,10)	<0.001
SOS	4 (2,5)	3 (2,5)	6 (4,7)	<0.001
qSOFA	1 (1,2)	1 (1,2)	2 (1,2)	<0.001
SIRS	2 (2,3)	2 (1,3)	2 (2,3)	0.017
ESI	2 (2,3)	2 (2,3)	2 (2,2)	0.001
Hemoculture status, n (%)				
Hemoculture positive	76 (13.8)	65 (13.1)	13 (18.8)	0.222
Gram positive cocci	26 (4.7)	19 (4.0)	7 (10.1)	0.024
Gram positive bacilli	1 (0.2)	0 (0)	1 (1.4)	0.008
Gram negative cocci	1 (0.2)	1 (0.2)	0 (0)	0.705
Gram negative bacilli	48 (8.7)	43 (8.9)	5 (7.2)	0.635
Secondary outcome, mean (SD)				
28-day intubation free day, day	28 (8.1)	28 (2.2)	4 (3.1)	<0.001
28-day vasopressor free day, day	28 (7.2)	28 (2.0)	6 (2.4)	<0.001
Source of infection, n (%)				
Pulmonary system	188 (34.2)	151 (31.4)	36 (52.2)	0.001
Urinary tract system	114 (20.7)	104 (21.6)	10 (14.5)	0.168
Gastrointestinal system	74 (13.5)	65 (13.5)	9 (13.0)	0.905
Cardiovascular system	3 (0.5)	2 (0.4)	1 (1.4)	0.278
Skin and soft tissue	40 (7.3)	34 (7.1)	6 (8.7)	0.634
Gynecologic system	2 (0.4)	2 (0.4)	0 (0)	0.592
Neurological system	7 (1.3)	6 (1.2)	1 (1.4)	0.892
Viral infection	38 (6.9)	37 (7.7)	1 (1.4)	0.055
Ear/nose/throat system	4 (0.7)	4 (0.8)	0 (0)	0.447
Unknown source of infection	70 (12.7)	65 (13.5)	5 (7.2)	0.142
CRBSI	9 (1.6)	9 (1.9)	0 (0)	0.252

*SD*, standard deviation; *IQR*, interquartile range; *AIDS*, acquired immunodeficiency syndrome; *ESRD*, end-stage renal disease; *RRT*, renal replacement therapy; *COPD*, chronic obstructive pulmonary disease; *ADL*, activities of daily living; *mm Hg*, millimeters of mercury; *mmol/dL*, millimoles per deciliter; *NEWS*, National Early Warning Score; *SOS*, Search Out Severity Score; *qSOFA*, quick Sequential Organ Failure Assessment; *SIRS*, Systemic Inflammatory Response Syndrome; *ESI*, Emergency Severity Index; *CRBSI*, catheter-related bloodstream infection.

**Table 2:** The area under the receiver operating characteristic curve with 95% confidence interval of predictive scores for predicting 28-day mortality, 28-day mechanical ventilator used, and 28-day vasopressor used.

Scores	AUROC (95% CI)		

	28-day mortality	28-day mechanical ventilator usage	28-day vasopressor usage
NEWS	0.770 (0.705, 0.835)	0.750 (0.700, 0.800)	0.763 (0.706, 0.819)
SOS	0.750 (0.685, 0.815)	0.751 (0.701, 0.801)	0.755 (0.697, 0.812)
qSOFA	0.747 (0.688, 0.806)	0.734 (0.689, 0.779)	0.741 (0.690, 0.791)
ESI triage	0.599 (0.542, 0.656)	0.642 (0.600, 0.683)	0.624 (0.576, 0.672)
SIRs	0.588 (0.522, 0.654)	0.581 (0.529, 0.632)	0.579 (0.521, 0.637)

*CI*, confidence interval; *AUROC*, area under the receiver operating characteristic curve; *NEWS*, National Early Warning Score; *SOS*, Search Out Severity Score; *qSOFA*, quick Sequential Organ Failure Assessment; *SIRS*, Systemic Inflammatory Response syndrome; *ESI*, Emergency Severity Index.

**Table 3:** Sensitivity and specificity for each predictive score for predicting 28-day mortality.

Score	News		SOS	
	Sensitivity	Specificity	Sensitivity	Specificity
≥1	98.55	2.49	97.10	3.95
≥2	95.65	6.86	95.65	12.68
≥3	94.20	15.38	89.86	27.65
≥4	91.30	28.69	85.51	49.90
≥5	86.96	45.74	68.12	70.89
≥6	78.26	61.12	53.62	83.99
≥7	72.46	74.64	36.23	91.89
≥8	56.52	85.24	23.19	96.05
≥9	40.58	90.23	11.59	97.71
≥10	27.54	96.05	1.45	99.38

Score	qSOFA		SIRS	
	Sensitivity	Specificity	Sensitivity	Specificity

≥1	94.20	24.53	95.65	5.61
≥2	69.57	74.84	85.51	25.78
3	17.39	96.47	49.28	65.07

Level	ESI Triage			
	Sensitivity		Specificity	

1	17.39		92.1	
2	89.96		26.82	
3	100		1.04	
4	100		0.21	

*NEWS*, National Early Warning Score; *SOS*, Search Out Severity Score; *qSOFA*, quick Sequential Organ Failure Assessment; *SIRS*, Systemic Inflammatory Response syndrome; *ESI*, Emergency Severity Index.
