# Supplementary material for: The Accuracy of Sepsis Screening Score for Mortality Prediction at Emergency Department Triage
Source: West J Emerg Med. 2022 Aug 11;23(5):698–705. doi: 10.5811/westjem.2022.6.56754 (PMC9541988; doi:10.5811/westjem.2022.6.56754)
Supplement: Supplementary file 1 [file wjem-23-698-s001.docx]

**Appendix 1** Parameters and values of each predictive scores in the study

| **NEWS** | | | | | | | | | | |
| --- | --- | --- | --- | --- | --- | --- | --- | --- | --- | --- |
| Score | RR | SpO2 | | Temperature | | SBP | | HR | Mental status | |
| 3 | <8 | <91 | | <35.0 | | <90 | | <40 |  | |
| 2 |  | 92-93 | |  | | 91-100 | |  |  | |
| 1 | 9-11 | 94-95 | | 35.1-36.0 | | 101-110 | | 41-50 |  | |
| 0 | 12-20 | >96 | | 36.1-38.0 | | 111-219 | | 50-99 | Alert | |
| 1 |  |  | | 38.1-39.0 | |  | | 91-110 |  | |
| 2 | 21-24 |  | | >39.1 | |  | | 111-130 |  | |
| 3 | >25 |  | |  | | >220 | | >131 | Confused,  Response to voice,  Response to pain,  Unresponsive | |
| **SOS** | | | | | | | | | | |
| Score | RR | | Temperature | | SBP | | HR | | Mental status | Urine* |
| 3 | <8 | |  | | <80 | | <40 | |  |  |
| 2 | ventilator | | <35.0 | | 81-90 | |  | |  | <500 ml/d |
| 1 |  | | 35.1-36.0 | | 91-100 | | 41-50 | | Confuse/Agitation | 501-999 ml/d |
| 0 | 9-20 | | 36.1-38.0 | | 101-180 | | 51-100 | | Alert | >1,000 ml/d |
| 1 | 21-25 | | 38.1-38.4 | | 181-199 | | 101-120 | | Response to voice |  |
| 2 | 26-35 | | >38.5 | | >200 | | 121-139 | | Response to pain |  |
| 3 | >36 | |  | | vasopressor | | >140 | | Unresponsive |  |
| **qSOFA** | | | | | | | | | | |
| Score | RR | | | | SBP | | | | Mental status | |
| 0 | <22 | | | | >100 | | | | Normal mental status | |
| 1 | >22 | | | | <100 | | | | Alter mental status | |
| **SIRS** | | | | | | | | | | |
| Score | RR | | Temperature | | HR | | WBC* | | | |
| 0 | <20 | | 36-38 | | <90 | | 4,000-12,000/mm^3^ | | | |
| 1 | >20 | | <36 or >38 | | >90 | | <4,000 or >12,000/mm^3^ or  >10% immature neutrophil | | | |
| **ESI triage** | | | | | | | | | | |
| Level | Description | | | | | | | | | |
| 1 | Require immediate Life-saving intervention | | | | | | | | | |
| 2 | High risk situation or confused/lethargic/disoriented or severe pain/distress | | | | | | | | | |
| 3 | Require many ED resources | | | | | | | | | |
| 4 | Require on ED resource | | | | | | | | | |
| 5 | Not require any ED resource | | | | | | | | | |

*not included in this study due to unavailability at the triage area
