# Supplementary material for: The Accuracy of Sepsis Screening Score for Mortality Prediction at Emergency Department Triage
Source: West J Emerg Med. 2022 Aug 11;23(5):698–705. doi: 10.5811/westjem.2022.6.56754 (PMC9541988; doi:10.5811/westjem.2022.6.56754)
Supplement: Supplementary file 2 [file wjem-23-698-s002.docx]

**Appendix 2** Sepsis or infection-related conditions (categorized by source of infection) according to ICD-10

| **Sepsis or infection-related conditions** | **ICD-10 diagnosis (Code)** |
| --- | --- |
| Sepsis | Sepsis (A41.9, A41.89); Severe sepsis without septic shock (R65.20); Severe sepsis with septic shock (R65.21) |
| Pulmonary system | Bacterial pneumonia (J13–J18); Acute bronchitis (J20); Acute bronchiolitis (J21); Unspecified acute lower respiratory infection (J22); Acute bronchitis (J20); Acute bronchiolitis (J21); Unspecified acute lower respiratory infection (J22); Abscess of lung and mediastinum; Pyothorax (J85, J86) |
| Urinary tract system | Acute pyelonephritis/pyonephrosis ((N10, N12, N136); Renal abscess (N151); Kidney infection, unspecified (N159); Acute cystitis (N300); Cystitis, unspecified (N308, N309); Urethritis and urethral abscess (N34); Urinary tract infections, unspecified (N390); Prostatitis and abscess of prostate (N41); Orchitis and epididymitis (N45); Catheter associated urinary tract infections (T835). |
| Gastrointestinal system | Acute appendicitis (K35); Abscess of anal and rectal regions, intestine, and liver (K61, K630, K750); Peritonitis (K65); Cholecystitis and cholangitis (K800, K801, K803, K804, K810, K819, K830); Intestinal infectious diseases (A00–A07, A09); Diverticulitis of intestine (K57); Noninfective gastroenteritis and colitis, unspecified (K529) |
| Cardiovascular system | Infective endocarditis (I33, T826); Infections due to cardiac and vascular devices (T827). |
| Skin and soft tissue | Infections of other skin and subcutaneous tissue including cellulitis, cutaneous abscess, furuncle, carbuncle, impetigo, acute lymphadenitis, folliculitis, mastitis (H050, J340, L00–L08, N61, T814); Pyogenic arthritis and prosthetic joint infection (M00, T845); Necrotizing fasciitis (M726); Infective myositis, synovitis and bursitis (M600, M650–M651, M710–M711); Osteomyelitis (M462–M465, M86); Infection due to internal prosthetic devices, implants and grafts (T857). |
| Gynecologic system | Pelvic inflammatory diseases (N70–N73, N751, N760–N764); Infections of genitourinary tract in pregnancy (O23) |
| Neurological system | Bacterial meningitis, encephalitis, and intracranial abscess (G00, G042, G049, G06). |
| Viral infection | Viral and other specified intestinal infections (A08); Viral pneumonia (J12) |
| Ear/nose/throat system | Suppurative and unspecified otitis media (H66); Streptococcal pharyngitis/tonsillitis (J020, J030); Acute pharyngitis/tonsillitis, unspecified (J029, J039); Scarlet fever (A38); Acute sinusitis (J01); Chronic sinusitis (J32); Deep neck space infections (J36, J390, J391); Mastoiditis (H70); Acute epiglottitis (J051); Infections of the jaws and mouth (K102, K122); Infections of the eye and adnexa (H00, H440); Infective otitis externa (H600–H603) |
| Unknown source of infection | Fever of unknown origin (R50) |
| CRBSI | Bloodstream infection due to central venous catheter. (T80.211) |
